# VRK1 Kinase Activity Modulating Histone H4K16 Acetylation Inhibited by SIRT2 and VRK-IN-1

**DOI:** 10.3390/ijms24054912

**Published:** 2023-03-03

**Authors:** Eva Monte-Serrano, Pedro A. Lazo

**Affiliations:** 1 Molecular Mechanisms of Cancer Program, Instituto de Biología Molecular y Celular del Cáncer, Consejo Superior de Investigaciones Científicas (CSIC)—Universidad de Salamanca, E-37007 Salamanca, Spain; 2 Instituto de Investigación Biomédica de Salamanca (IBSAL), Hospital Universitario de Salamanca, E-37007 Salamanca, Spain

**Keywords:** VRK1, VRK-IN-1, SIRT2, DNA damage response, histone H4, acetylation, Tip60, KAT5

## Abstract

The accessibility of DNA to different cellular functions requires a dynamic regulation of chromatin organization that is mediated by different epigenetic modifications, which regulate chromatin accessibility and degree of compaction. These epigenetic modifications, particularly the acetylation of histone H4 in lysine 14 (H4K16ac), determine the degree of chromatin accessibility to different nuclear functions, as well as to DNA damage drugs. H4K16ac is regulated by the balance between two alternative histone modifications, acetylation and deacetylation, which are mediated by acetylases and deacetylases. Tip60/KAT5 acetylates, and SIRT2 deacetylates histone H4K16. However, the balance between these two epigenetic enzymes is unknown. VRK1 regulates the level of H4K16 acetylation by activating Tip60. We have shown that the VRK1 and SIRT2 are able to form a stable protein complex. For this work, we used in vitro interaction, pull-down and in vitro kinase assays. In cells, their interaction and colocalization were detected by immunoprecipitation and immunofluorescence. The kinase activity of VRK1 is inhibited by a direct interaction of its N-terminal kinase domain with SIRT2 in vitro. This interaction causes a loss of H4K16ac similarly to the effect of a novel VRK1 inhibitor (VRK-IN-1) or VRK1 depletion. The use of specific SIRT2 inhibitors in lung adenocarcinoma cells induces H4K16ac, contrary to the novel VRK-IN-1 inhibitor, which prevents H4K16ac and a correct DNA damage response. Therefore, the inhibition of SIRT2 can cooperate with VRK1 in the accessibility of drugs to chromatin in response to DNA damage caused by doxorubicin.

## 1. Introduction

Dynamic chromatin relaxation and remodeling are associated with basic nuclear functions, normal or pathological. The epigenetic modification of histones and its patterns determine the roles chromatin remodeling plays. A specific histone epigenetic mark underlying chromatin relaxation is the acetylation of histone H4 in K16 (H4K16ac), which is essential for chromatin protein interactions in different pathways [1]. H4K16ac can facilitate DNA damage because of the DNA accessibility to oxidative stress or other genotoxic agents. Additionally, this epigenetic modification participates in the initial recruitment of sequential DNA repair proteins. H4K16ac is associated with several processes that require a dynamic local relaxation and opening of chromatin, such as gene transcription [2], recombination [3,4] or DNA damage responses [5,6], as well as differentiation and cell death [7], which reflects the complexity of its regulation. The enzymes performing histone epigenetic modifications can be potentially targeted as part of a therapeutic strategy [8,9,10], particularly if they can be used in synthetic lethality strategies [11].

The balance between acetylated and deacetylated H4 in K16 is regulated by the coordination between histone acetyl transferases (HAT/KAT), such as Tip60/KAT5 [12], and histone deacetylases (HDAC), such as SIRT2 [13,14,15]. However, the mechanism by which these two types of histone-modifying epigenetic enzymes and their coordination are regulated is unknown. One potential mechanism is that KAT5 and HDACs, SIRT1 and SIRT2 (sirtuins 1 and 2) are coordinated by members of other enzyme families, such as kinases, or alternatively by specific protein interactions that regulate the balance of local histone acetylation and their functional roles, like transcription, replication or pathological roles, such as DNA damage responses (DDR). In this context, the chromatin kinase VRK1 is a potential coordinating protein that regulates chromatin relaxation and accessibility [16]. 

Histone deacetylases (HDAC) are dysregulated in many cancer types, and their inhibition is a potential candidate for a novel therapeutic strategy [17,18]. Inhibition of HDAC will cause an accumulation of histone acetylation that is associated with a more relaxed and accessible chromatin to DNA damage. For this reason, the accumulation of this histone mark sensitizes cells to radiotherapy or genotoxic drugs used in cancer treatments [19,20], since its irreversibility can prevent progression of the repair process. 

SIRT2 is an HDAC that regulates cell cycle progression and genome stability [21]. Similarly, the nuclear and chromatin kinase VRK1 regulates cell cycle progression [22,23] and genome stability [24,25]. Moreover, it has been shown that VRK1 directly interacts and phosphorylates Tip60/KAT5, leading to its stabilization and translocation to chromatin where acetylates H4 in K16 [26,27]. Furthermore, VRK1 also plays several roles in the response to gene transcription [28,29] and DNA damage responses, processes which require a local and dynamic coordination of chromatin reorganization [30,31,32]. For this reason, the levels of H4K16 acetylation regulate chromatin accessibility as a result of the balance between the chromatin kinase VRK1 and SIRT2. 

## 2. Results

### 2.1. SIRT2 Inhibition and Its Combination with Doxorubicin Facilitate Histone H4K16 Acetylation

**Figure 1:**
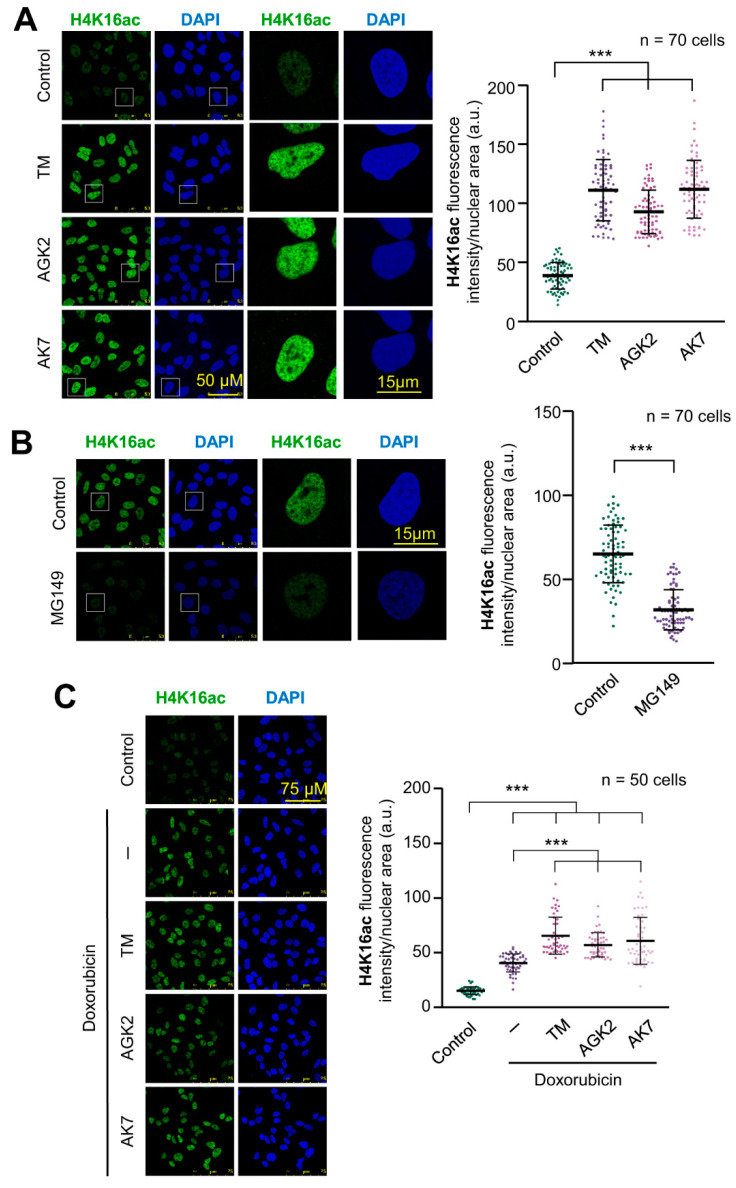
SIRT2 and KAT inhibitors have opposite effects on H4K16 acetylation. (**A**) Accumulation of H4K16 acetylation in A549 cells that were serum-deprived for 48 h and treated with three histones deacetylase inhibitors, 5 µM thiomyristoyl (TM), 5 µM AGK2 or 8 µM AK7. The effect was determined by immunofluorescence. The quantification is shown to the right. (**B**) Effect of the KAT inhibitor MG149 (1 µM) on the levels of H4K16 acetylation in A549 cells. The effect was determined by immunofluorescence. The quantification is shown to the right. (**C**) Effect of treating A549 cells with combinations of 3 µM doxorubicin and SIRT2 inhibitors. The effect was determined by immunofluorescence. The quantification is shown to the right. *** *p* < 0.001.

Chromatin relaxation is associated with H4K16 acetylation and facilitates the access of proteins participating in DNA damage response to chromatin. SIRT2 (sirtuin 2) and Tip60/KAT5 have opposite roles in histone H4 acetylation, and the balance between these two enzymes determines the acetylation state of genomic regions, and their accessibility to DNA. Initially, the effect of three SIRT2 inhibitors, thiomyristoyl (TM), AGK2 and AK7 that block histone deacetylation, were tested individually on the basal levels of H4K16ac. Individually, each of these inhibitors caused a very significant increase in the levels of H4K16ac in A549 cells (Figure 1A). The opposite effect, a reduction in H4K16ac levels, was detected by the inhibition of Tip60/KAT5 with MG149, which inhibits its acetyltransferase activity (Figure 1B). The treatment of cells with doxorubicin, an intercalating DNA drug, caused an increase in histone H4K16 acetylation due to the activation of Tip60/KAT5 by VRK1 [26,27]. When doxorubicin was combined with any of the three SIRT2 inhibitors, it resulted in a higher accumulation of this histone mark because of the inhibition of SIRT2 deacetylase activity [26,27] (Figure 1C, Supplementary Figure S1). The increase in H4K16ac in response to doxorubicin treatment was impaired by either the inhibition of Tip60 with MG149 or by VRK1 depletion that prevents the activation of Tip60/KAT5 (Supplementary Figure S2) [26,27]. Thus, the three SIRT2 inhibitors (TM, AGK2, AK7) cause a strong increase in H4K16ac, which are even higher than those of doxorubicin treatment by itself. 

### 2.2. VRK1 Directly Interacts with SIRT2 In Vitro and In Vivo

**Figure 2:**
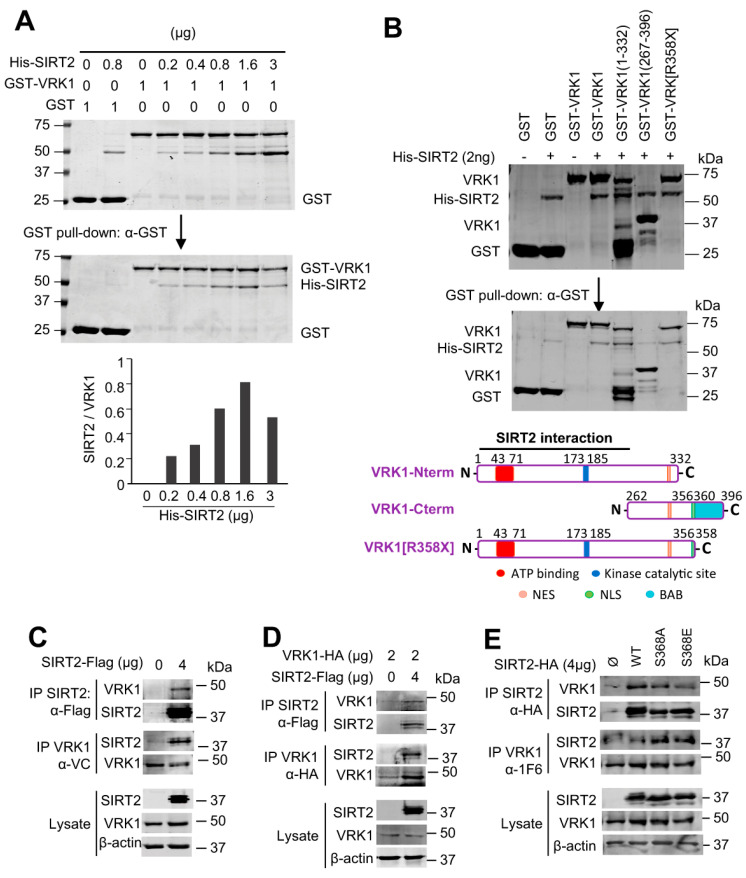
SIRT2 directly interacts with VRK1 in vitro and in vivo. (**A**) Concentration dependent in vitro interaction between bacterially expressed and purified GST-VRK1 protein and increasing amounts of purified His-SIRT2 in a GST pull-down in vitro assay. At the bottom, the quantification of the interaction is shown. GST-VRK1 and the indicated amounts of His-SIRT2 were mixed and incubated overnight at 4 °C. GST-VRK1 pull-down was performed using anti-GST antibody. Empty-GST protein was used as a negative control. The upper WB shows the control loading proteins. (**B**) Detection of the VRK1 region of interaction with SIRT2. Different GST-VRK1 constructs were used in the pull-down assay as described in A. NES: nuclear export signal. NLS: nuclear localization signal. BAB: basic-acid-basic motif. At the bottom, a diagram indicating the location of the interaction region in the VRK1 protein is shown. (**C**) Interaction of endogenous VRK1 with a transfected and tagged SIRT2-flag plasmid in HEK293T cells. The interaction was detected by reciprocal immunoprecipitations with anti-VRK1 and anti-FLAG rabbit antibodies shown at the bottom. (**D**) Interaction of VRK1-HA and SIRT2-flag expressed from plasmids transfected in HEK293T cells that were detected in reciprocal immunoprecipitations with anti-HA and anti-FLAG rabbit antibodies. In the lysate, VRK1 was detected with an anti-VRK1 antibody. (**E**) Interaction of endogenous VRK1 and SIRT2-HA from transfected and tagged plasmids mutated in its known S368 phosphorylation site in HEK293T cells. Interactions were detected in reciprocal immunoprecipitations with anti-VRK1 and anti-HA rabbit antibodies.

Acetylation of histone H4K16 is reversible. Therefore, it is likely that an unidentified mechanism might regulate the coordination and balance between HDAC and Tip60/KAT5 enzymes, which have opposite activities. In this context, a kinase, such as the chromatin kinase VRK1, is a likely candidate. H4K16 acetylation is mediated by Tip60/KAT5, which is regulated by VRK1 though a specific activating phosphorylation of Tip60 in T158 [26,27]. SIRT1 and SIRT2 deacetylate H4K16ac [13,15]. Therefore, it was studied whether VRK1 and SIRT2 are able to form a stable protein complex. For this aim, we first determined the in vitro interaction between tagged GST-VRK1, and SIRT2-his, using bacterially expressed and purified proteins, which can detect a direct and stable protein interaction. SIRT2 directly and stably interacted with VRK1 in a dose-dependent manner (Figure 2A). Next, to identify the VRK1 region of interaction, several GST-VRK1 constructs spanning different regions of VRK1 were expressed in bacteria, and purified fusion proteins were used in pull-down assays with SIRT2-his as the target (Figure 2B). The common VRK1 region of interaction corresponds to residues 1–262, which comprise the kinase domain, and includes both the ATP binding site and the catalytic site. However, SIRT2 did not interact with the low complexity C-terminal VRK1 regulatory domain (residues 267–396) (Figure 2B). To confirm the VRK1-SIRT2 interaction in vivo, HEK293T cells were transfected with tagged SIRT2-Flag, which was able to interact with the endogenous VRK1 in reciprocal immunoprecipitation experiments (Figure 2C, top panel). The immunoprecipitation of the endogenous VRK1 protein with an antibody targeting its C-terminus [33] confirmed that the VRK1 C-terminus is not involved in the interaction, and thus the N-terminus is available for recognition and interaction with SIRT2-Flag (Figure 2C, center panel). The colocalization of VRK1 and SIRT2 in nuclei was confirmed by immunofluorescence in A549 cells (Supplementary Figure S3). This VRK1-SIRT2 interaction was further confirmed when cells were transfected with both tagged proteins and detected in reciprocal immunoprecipitation experiments (Figure 2D). Furthermore, the VRK1-SIRT2 interaction is independent of the SIRT2-S368 mutation to either Ala or Glu (Figure 2E), a known phosphorylation site of SIRT2 in cell cycle progression [34,35]. 

### 2.3. SIRT2 Inhibits the Kinase Activity of VRK1

**Figure 3:**
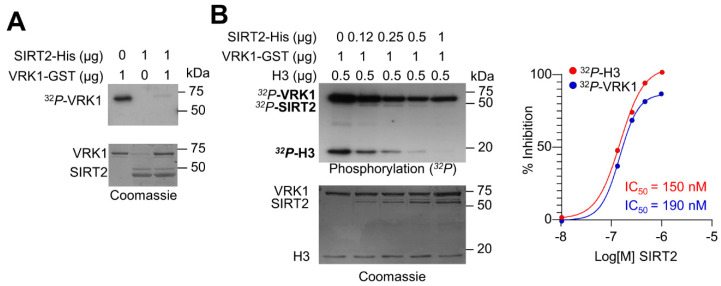
Inhibition of VRK1 kinase activity by SIRT2. (**A**) In vitro kinase assay to detect the phosphorylation of VRK1 and SIRT2 with purified proteins. SIRT2 inhibits the autophosphorylation of VRK1. The exposure time to detect the autophosphorylation VRK1 is two hours. (**B**) SIRT2 dose dependent inhibition of the kinase activity of VRK1 in autophosphorylation and transphosphorylation with histone H3 as substrate. The exposure time to detect the autophosphorylation VRK1 and H3 phosphorylation is forty-eight hours. To the right, the IC50 for both activities is shown.

Because VRK1 and SIRT2 have opposite roles on the acetylation of histone H4 in K16, it is likely that there is between these two enzyme activities. Therefore, we tested whether, as a result of the VRK1-SIRT2 interaction, the VRK1 activity could be altered, and thus permit the deacetylation of H4K16 mediated by SIRT2. This is a likely possibility since SIRT2 interacts with the catalytic domain of VRK1. For this aim, we performed an initial in vitro experiment with both proteins expressed and purified in bacteria. VRK1 by itself has a strong autophosphorylation activity that was inhibited in the presence of SIRT2 (Figure 3A). Next, we tested different concentrations to detect both the inhibitory effect of SIRT2 on VRK1 autophosphorylation, and on H3 phosphorylation, which is a direct target of VRK1 [24,36,37,38]. SIRT2 inhibited both the VRK1 autophosphorylation as well as the phosphorylation of histone H3 in a dose-dependent manner with an IC50 of 190 nM and 150 nM, respectively (Figure 3B).

### 2.4. The VRK-IN-1 Inhibitor Impairs the Phosphorylation of Histone H3 and p53

**Figure 4:**
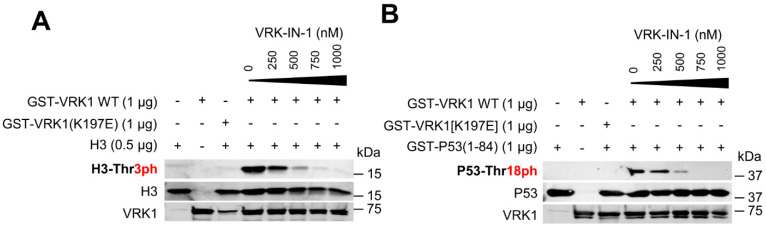
VRK-IN-1 inhibitor blocks the phosphorylation of histone H3 (**A**) and p53 (**B**) by VRK1. (**A**) GST-VRK1 and human histone H3 were incubated with increasing concentrations of the VRK-IN-1 inhibitor (0–1 µM) for 2 h at 37 °C. Then, an in vitro kinase assay was carried out and phospho-specific signal were identified by WB with H3T3Ph antibody. As a negative control, a kinase-dead VRK1 (K179E) was used. (**B**) GST-VRK1 and GST-P53(1-84) were incubated with increasing concentrations of the VRK-IN-1 inhibitor (0–1 µM) for 2 h at 37 °C. Consecutively, an in vitro kinase assay was performed and phospho-specific signals were detected by WB with P53T18Ph antibody. As a negative control, a kinase-dead VRK1 (K179E) was used.

The levels of H4K16 acetylation are regulated by VRK1 [26,27]. In order to manipulate the activity of VRK1 function, the development of specific inhibitors is necessary. VRK1, because of its structural characteristics, is not inhibited by current inhibitors targeting different kinase families of the human kinome [39,40]. VRK-IN-1 is a novel inhibitor recently developed with a structure based on an aminopyridine scaffold that has a high affinity for VRK1, and to a lesser extent for VRK2 [41,42]. First, we tested the effect of the VRK1-IN-1 inhibitor in an in vitro kinase assay using two of the known protein phosphorylation targets of VRK1, histone H3 [38] and p53 [43,44]. The VRK1-IN-1 inhibitor blocked the specific phosphorylation of histone H3 in Thr3 (Figure 4A) and of p53 in Thr18 (Figure 4B) with an IC50 of 250 and 340 nM, respectively. These data indicated that this novel VRK-IN-1 inhibitor has potential for its pharmacological development and improvement. 

### 2.5. The VRK-IN-1 Inhibitor Facilitates the Accumulation of Endogenous DNA Strand Breaks

**Figure 5:**
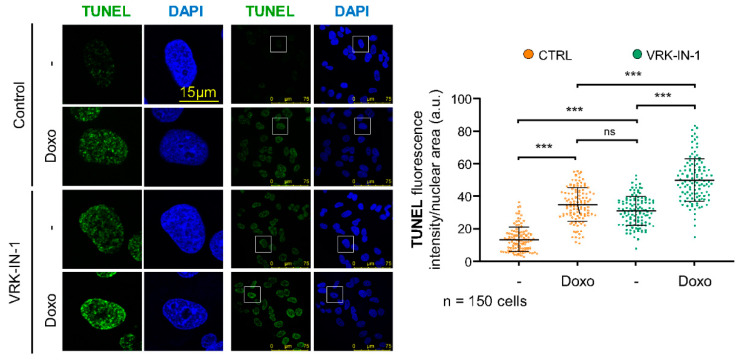
VRK-IN-1 treatment induces an accumulation of 3′-OH DNA ends resulting from DNA strand-breaks. A549 cells were treated with 600 nM VRK-IN-1 for 24 h and 3 µM doxorubicin for 2 h. The available free 3′-DNA ends were detected by labeling with terminal deoxynucleotidyl transferase (TdT) in TUNEL assays. The quantification is shown to the right. ns: not significant, *** *p* < 0.001. -: not treated; Doxo: doxorubicin.

Most of the endogenous DNA damage is the result of oxidative stress, which is very effectively repaired by OGG1 [45]. In these endogenous oxidative DNA lesions that are not repaired, there is an increase in single-strand breaks, which can be detected by labelling the free 3′-DNA ends in broken strands with TdT using TUNEL assays. VRK1 depletion causes an increase in free DNA-ends [46]. Therefore, we tested whether the VRK-IN-1 inhibitor could have the same effect on the level of DNA damage cause by doxorubicin (Figure 5). A549 cells treated with doxorubicin showed an increase of free DNA-ends, and the VRK-IN-1 inhibitor cause similar level of DNA damage. Moreover, the combination of doxorubicin and VRK-IN-1 caused a significant increment in the levels of free-DNA ends (Figure 5). This result suggested that the inhibition of VRK1 combined with DNA damaging agents can promote tumor cell death.

### 2.6. The VRK-IN-1 Inhibitor Reduces H4K16 Acetylation Levels

**Figure 6:**
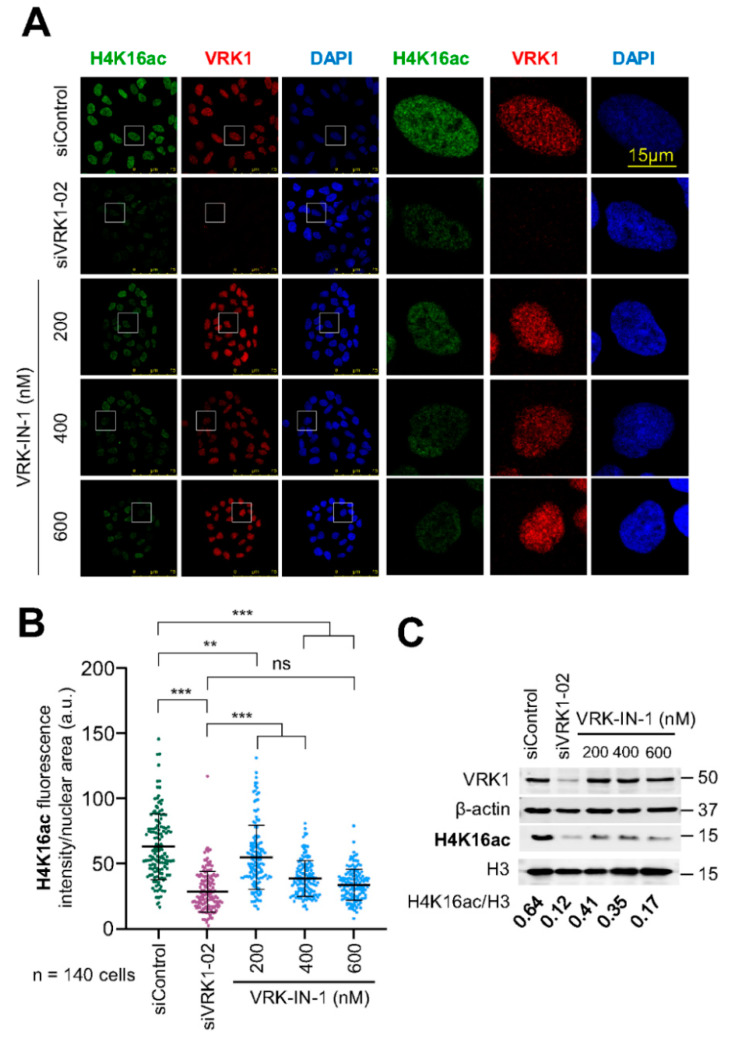
VRK1 depletion or inhibition with VRK-IN-1 reduces H4K16 acetylation levels in vivo. VRK1 was knocked down using a specific siRNA (siVRK1-02). A549 cells were deprived of serum for 48 h and incubated with different concentrations of VRK-IN-1 as indicated for 24 h. (**A**) Image panels showing the levels of H4K16 acetylation after VRK1 depletion or VRK-IN-1 treatment. The effect was detected by immunofluorescence with a specific antibody anti-H4K16ac. The VRK-IN-1 inhibitor was used at the indicated doses. The box indicates the individual cell shown to the right for detail. (**B**) Quantification of H4K16ac fluorescence. (**C**) Immunoblots show the levels of H4K16ac of acidic extracts and VRK1 for knock-down control. β-actin and histone H3 were used as a loading control. In the bottom of WB, the H4K16ac levels per H3 quantification are shown. ns: not significant, ** *p* < 0.01, *** *p* < 0.001.

VRK1 controls the acetylation of H4K16 by regulating the translocation of Tip60 from the nucleoplasm to chromatin and activating the Tip60 trans-acetylase activity in non-dividing cells [26,27]. Therefore, we tested the effect of different concentrations of the VRK-IN-1 inhibitor on the endogenous basal levels of H4K16 acetylation. For this aim, serum-deprived A549 cells were incubated in the presence of different concentrations of the VRK-IN-1 inhibitor for twenty-four hours, and the levels of H4K16ac was determined by immunofluorescence and immunoblots. The VRK-IN-1 inhibitor resulted in the loss of H4K16 acetylation (Figure 6). This effect of the VRK-IN1 inhibitor mimics the effect of VRK1 depletion on H4K16 acetylation (Supplementary Figure S1) [26].

### 2.7. The VRK-IN-1 Inhibitor Impairs the DNA Damage Response Induced by Doxorubicin

**Figure 7:**
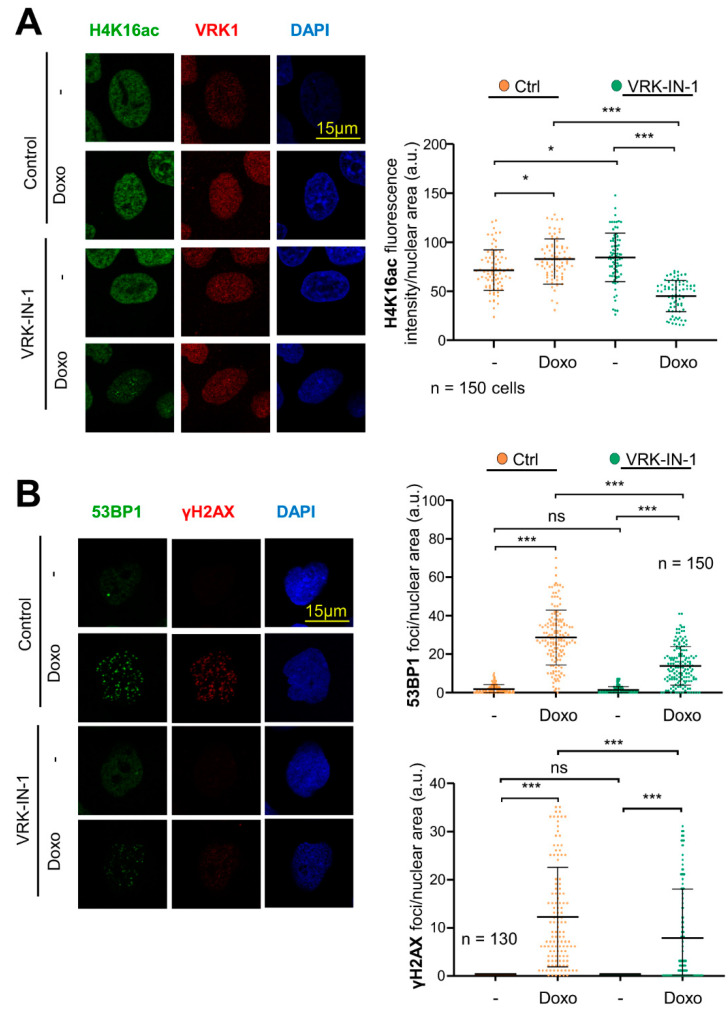
VRK-IN-1 treatment impairs H4K16 acetylation and 53BP1 and γH2AX foci formation in response to DNA damage caused by doxorubicin. Serum-deprived A549 cells were treated with 600 nM VRK-IN-1 for 24 h. DNA damage was induced by 3 µM of doxorubicin for 2 h. (**A**) Effect of VRK-IN-1 on the levels of H4K16ac induced by doxorubicin. Image panels showing H4K16ac levels detected by immunofluorescence with a specific antibody anti-H4K16ac. The field image is shown in Figure S4A. (**B**) Effect of VRK-IN-1 on the induction of 53BP1 and γH2AX foci induced by doxorubicin. Image panels showing 53BP1 and γH2AX foci levels detected by immunofluorescence with a specific antibody anti-53BP1 and anti-γH2AX. The field image is shown is Figure S4B. The quantification is shown to the right. ns: not significant, * *p* < 0.05, *** *p* < 0.001. -: not treated; Doxo: doxorubicin.

The use of VRK1 inhibitors, which should prevent the activation of Tip60 by VRK1 and avoid the recruitment of DNA repair proteins, should cause an increase in the accumulation of DNA damage, by maintaining a local open chromatin organization and impairing DDR progression. Therefore, we studied the effect of the VRK-IN-1 inhibitor to determine its effect on the accumulation of DNA damage induced by doxorubicin, which was determined by the level of H4K16ac reflecting the early response to damage mediated by Tip60, and γH2AX and 53BP1 foci that reflect DNA damage. The VRK-IN1 inhibitor reduced the level of H4K16ac (Figure 7A), which indicates that the activation of Tip60 was impaired, and thus unable to recruit repair proteins. Next, we determined that VRK-IN-1 reduced both the formation of γH2AX and 53BP1 foci induced in response to doxorubicin (Figure 7B), indicating that the activation of the NHEJ repair pathway was defective.

## 3. Discussion

The control of chromatin relaxation is necessary to facilitate different processes that require very specific and sequential regulatory mechanisms, which will be adapted to the specific need of a particular chromatin region. These local chromatin relaxations are critical for the correct functioning of the cellular processes requiring a dynamic chromatin remodeling, transcription, replication or DNA repair, in this latter case from DNA damage that locally alters chromatin. However, this relaxation is selective and transient for its specific temporal function. Thus, it requires a very tight regulation, and coordination, of the histones posttranslational modifications that are implicated, as is the case of H4K16 acetylation. H4K16ac is required for the recruitment of different proteins involved in the sequential specific steps in DDR pathways. An excess of H4K16 acetylation or its persistence in time will facilitate the chromatin accessibility to genotoxic agents, and if it is not removed, it might interfere with the progression and recruitment of other proteins in the DNA repair process. Therefore, this H4 epigenetic modification requires a precise regulation of its levels in time and space, which implicates several types of enzymes, including an acetyl transferase, such as Tip60/KAT5, and a histone deacetylase such as SIRT2, or another HDAC member. Moreover, the balance between these two enzyme activities, with opposite effects, requires a coordinator. The most suitable candidate for such coordination is a nuclear kinase, such as VRK1, which is also known as nucleosomal kinase-1 (NHK-1) in *Drosophila melanogaster* [47]. We have identified a mechanism in which there can be a crosstalk between the two activities, acetylase and deacetylase. VRK1 activates Tip60 that acetylates H4 in K16 [26,27], but when SIRT2 interacts with VRK1, its kinase activity is inhibited, and permits the histone deacetylation mediated by SIRT2. 

The kinase activity of VRK1 can be manipulated by novel inhibitors, such as VRK-IN-1 [41,42], the first inhibitor in its class, and can inhibit VRK1 kinase activity at nanomolar concentrations, and thus can be the base for future pharmacological development. We have shown that this inhibitor impairs the activity of VRK1 on two of its known substrates in vitro, histone H3 and p53. Additionally, this inhibitor impairs the acetylation of H4K16, and thus DNA repair enzymes cannot be recruited, facilitating the accumulation of DNA damage.

**Figure 8:**
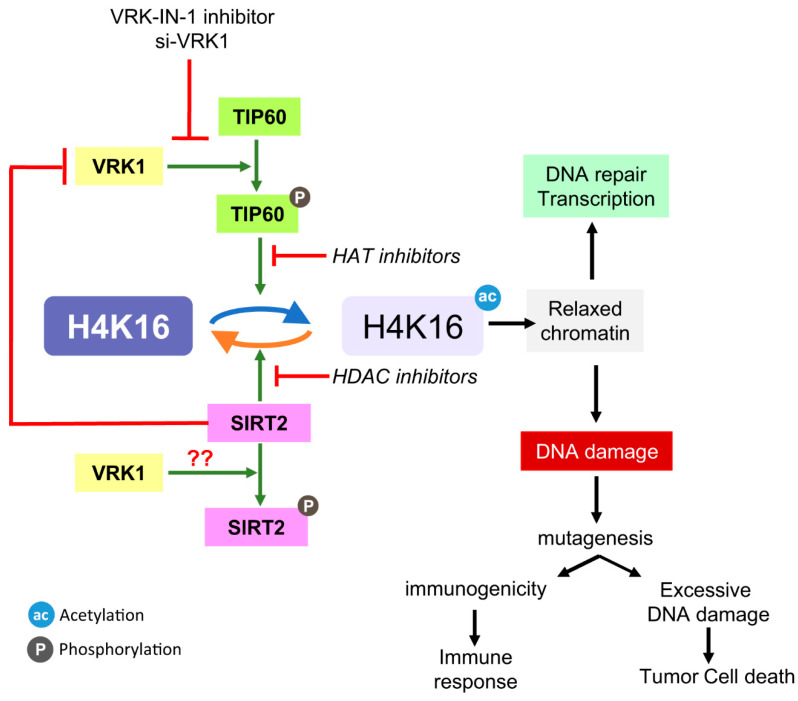
Regulation of the level of H4K16 acetylation. Diagram of the interplay between SIRT2 and Tip60/KAT5 regulating the level of H4K16 acetylation and their manipulation by different types of inhibitors targeting different enzymes. HAT inhibitor: MG149; SIRT2 inhibitors: Thiomyristoyl, AK7, AGK2; SIRT1 inhibitor. ac: acetylation; ph: phosphorylation.

In this report we have identified a novel mechanism coordinating the level of H4K16ac. This mechanism (Figure 8) implicates the nuclear VRK1 chromatin kinase [26]. This kinase modulates the activity of the Tip60/KAT5 acetylase as well as that of the SIRT2 histone deacetylase. However, the mechanisms are different depending on the enzyme. VRK1 directly phosphorylates Tip60, leading to its translocation to chromatin and activating its acetylase activity [26,27]. Nevertheless, the switch off is mediated, not by a phosphorylation, but by a direct protein interaction with the SIRT2 deacetylase. When VRK1 interacts with SIRT2, the kinase activity of VRK1 is inhibited, and consequently SIRT2 can perform the deacetylation at the same time that acetylation is suppressed. Functionally, the loss of Tip60/KAT5 activation by VRK1 will result in an impaired recruitment of sensor and repair proteins, while the accumulation of H4K16ac, as a result of using SIRT2 or VRK1 inhibitors, will prevent progression of the DNA repair pathway, because the dynamic regulation is blocked by maintaining H4K16 in an acetylated state.

The involvement of three enzymes, VRK1, Tip60/KAT5 and SIRT2, in the regulation of the level of acetylation of H4K16 opens up the possibility of its pharmacological manipulation by their combination, to promote the elimination of tumor cells. The persistence of an open chromatin will facilitate the access to genotoxic agents such as oxidative stress of chemotherapeutic drugs, and these will promote the accumulation of DNA damage and compromise tumor cell viability. An alternative effect, as a consequence of increased genetic damage, is the stimulation of an immunogenic response against new tumor antigens that may contribute to the elimination of tumor cells [48,49].

## 4. Materials and Methods

### 4.1. Reagents and Inhibitors

AGK2, AK7, thiomyristoyl and selisistat were purchased from Selleckchem (Houston, TX, USA); MG149 from Axon MedChem (Groningen, The Netherlands); doxorubicin hydrochloride from Sigma-Aldrich (St. Louis, MO, USA); and VRK-IN1 from MedChemExpress (Monmouth Junction, NJ, USA). Cells were treated following the indicated schemes (Table 1).

### 4.2. Plasmids

Plasmid pCEFL-HA-VRK1 was used to express human VRK1 [32,50] and pcDNA3.1-Flag-SIRT1 and pcDNA3.1-Flag-SIRT2 plasmids were used to express human SIRT1 and SIRT2 [13,51]. GeneJET Plasmid Maxiprep kit (Thermo Fisher Scientific, Waltham, MA, USA) was used for plasmid purifications.

For in vitro kinase assays, PGEX-4T-VRK1-GST, pGEX-4T-VRK1[K179E]-GST, pGEX-2T-p53[1-84]-GST and pET30a-SIRT2-6xHis plasmids [13,46,50] were used for expression and purification of the fusion proteins expressed in *Escherichia coli* strain BL21. Protein expressions were induced with IPTG 0.2 M 37 °C for 2 h and bacteria were lysed with lysis buffer (20 mM Tris HCl pH 8.0, NaCl 500 mM, 1% Triton X-100, 0.025% NaN_3_, 0.2 μg/mL lysozyme and 5 mM DTT) or BC-500 buffer (20 mM Tris pH 8.0, 100 mM NaCl, 10 mM EDTA pH 8.0, 0.1% NP40 and 2% sarkosyl). The resulting GST or His fusion proteins were incubated with Glutathione Sepharose 4B beads (GE Healthcare Systems; Chicago, IL, USA) or NiNTA Agarose beads (Qiagen; Hilden, Germany), respectively. After several washes with the corresponding lysis buffer, the proteins were obtained by elution with glutathione 20 mM or imidazole 50 mM. Purified proteins were aliquoted and stored at −80 °C.

### 4.3. Cell Lines, Culture and Transfections

The following validated cell lines HEK 293T (CRL-3216), A549 (CCL-185) and U2OS (HTB-96) were obtained from the American Type Culture Collection (ATCC), and were mycoplasma free. Cells were cultured in DMEM (Gibco-Life Technologies Invitrogen; Waltham, MA, USA) supplemented with 10% fetal bovine serum (FBS), 2 mM glutamine (L-glutamine) and 1% penicillin-streptomycin (Pen/Strep), all obtained from Gibco-Life Technologies (Waltham, MA, USA). For the purpose of experiments, cells were grown to 80% confluence. Cells were washed with PBS and detached using TrypLE-Express (Gibco-Life Technologies-Invitrogen; Waltham, MA, USA). Serum starvation (DMEM supplemented with 0.5% FBS, 2 mM L-glutamine, 1% Pen/Strep) was performed for 48 h when indicated.

Plasmid transfections were performed as previously reported [26]. Briefly, 4–6 µg DNA was diluted in polyethylenimine (PEI; Polysciences; Warrington, PA, USA) reagent and incubated for 30 min. DNA-PEI mix was added by gently pipetting dropwise to the cells, which were assayed 48 h after transfection.

### 4.4. VRK1 Depletion by siRNA

Si-RNA was used for depletion of VRK1. The VRK1 sequences targeted by these siRNA from Dharmacon were 5′-CAAGGAACCTGGTGTTGAA-3′ (siVRK1-02), and 5′-GGAAUGGAAAGUAGGAUUA-3′ (siVRK1-03). ON-TARGET plus siControl non-targeting siRNA (siControl) was used as a negative control. Lipotransfectin (Solmeglas; Madrid, Spain) was diluted in Opti-MEM (GIBCO-Life Technologies) according to manufacturer guidelines. siRNA (200 nM) was diluted in Opti-MEM and added to the lipotransfectin-Opti-MEM mix. After 30 min of incubation, the lipotransfectin-Opti-MEM-RNA mix was added by gently pipetting dropwise to the cells. Cells were maintained with antibiotic-free media 72 h after siRNA transfection, as previously reported [52].

### 4.5. Cell Lysates and Acidic Histone Extraction

All steps used for protein extraction were performed in ice. Cell lysates were prepared by suspending cells in lysis buffer (50 mM Tris HCl pH 8.0, 150 mM NaCl, 1% triton X-100 and 1 mM EDTA) supplemented by phosphatases inhibitors (1 mM sodium fluoride and 1 mM sodium orthovanadate) and proteases inhibitors (1 mM PMSF, 10 mg/mL aprotinin, and 10 mg/mL leupeptin). The suspension was incubated at 4 °C for 15 min followed by centrifugation (16,000× *g*, 15 min, 4 °C). Histones were isolated by acidic extraction, as previously described [53]. Protein concentration was determined using the BCA protein assay kit (Thermo Fisher Scientific; Waltham, MA, USA). Forty micrograms of protein was used for immunoblots; 5–10 µg of acidic extracts of histones were used for immunoblots.

### 4.6. Antibodies

Antibodies used in this study, applications and conditions are listed in Table 2. They were diluted in TBS-T buffer (25 mM Tris HCl pH 8.0, 50 mM NaCl and 2.5 mM KCl, 0.1% Tween-20) or PBS-1% BSA for immunoblots or immunofluorescence assays, respectively.

### 4.7. Immunoprecipitations

Immunoprecipitations were performed using 0.5–1 mg of protein from cell lysates. Protein extracts were incubated with the corresponding antibody for each experiment for 6–8 h at 4 °C in rotation. Subsequently, 40 µL of Protein G–Agarose Resin 4 Rapid Run (4RRPG, Agarose Bead Technologies; Madrid, Spain) was added to the protein-antibody immune complexes overnight at 4 °C on a rotating wheel^5^. The immunoprecipitated was collected by centrifugation (500× *g*, 2 min, 4 °C) and washed three times with lysis buffer [52,55].

### 4.8. Immunoblots

Lysates and immunoprecipitates were boiled at 95 °C for 5 min in sample loading buffer (62.5 mM Tris-HCl pH 6.8, 10% glycerol, 2.3% SDS, 0.1% bromophenol blue and 5% β-mercaptoethanol). After separation via SDS-PAGE, proteins were transferred to PVDF Immobilon-FL membranes (0.22 or 0.45 µm pore size; Millipore; Burlington, MA, USA) [26,55]. Membranes were blocked for 1 h at room temperature with 5% nonfat milk or 5% of BSA in TBS-T buffer. Next, membranes were washed 3 times for 10 min each time in TBS-T and incubated with the primary antibody overnight at 4 °C. Next day, after three washes of 10 min in TBS-T buffer, membranes were incubated in the darkness with their corresponding secondary antibodies (Table 3) diluted 1:10,000 in TBS-T for 1 h. Membranes were washed three more times in TBS-T for 10 min. Finally, fluorescence signals were detected using a LI-COR Odyssey Infrared Imaging System (LI-COR Biosciences; Lincoln, NE, USA). Densitometric analysis of Western blots were performed using ImageJ software (version 1.53e). All Western blots were performed in triplicate and correspond to the accompanying immunofluorescence image.

### 4.9. Immunofluorescence and Confocal Microscopy

Cells were cultured with glass coverslips (Thermo Fisher Scientific; Waltham, MA, USA) in the culture dishes as previously described. After the corresponding times and treatments, cells were fixed with 3% paraformaldehyde (PFA) in PBS for 15 min, and treated with 200 mM glycine solution to eliminate the PFA. Cells were permeabilized with 0.2% triton X-100 for 15 min and blocked with PBS-1% BSA with 0.1% sodium azide for 1 h at room temperature, or overnight at 4 °C [32,56]. Coverslips were consecutively incubated with two primary antibodies for concurrently protein detection. The primary antibodies were incubated between 56 h at room temperature or overnight at 4 °C. Afterwards, cells were washed with PBS 3 times and incubated with the secondary antibodies (Table 3) at 1:1000 dilution for 1 h at room temperature in the dark. All next steps were carried out in darkness. After 3 more washes with PBS, nuclei were stained with DAPI (4′, 6diamidino-2-phenylindole) at 1:1000 dilution for 5 min, followed by three washes with PBS. Coverslips were mounted with a drop of mounting medium (MOWIOL) in microscope slides. Cell Images were captured with a LEICA SP5 DMI-6000B confocal microscope (Leica; Wetzlar, Germany), with the following lasers: Argon (488 nm), DPSS (561 nm) and UV Diode (405 nm). These images were acquired with a 63.0× lens zoomed in 1.5× with a 1024 × 1024 frame and 600 Hz scanning speed. Images were analyzed with ImageJ (version 1.53e) software (https://imagej.nih.gov/ij). These imaging experiments were independently performed three times.

### 4.10. TUNEL Assay

Accessible 3′-OH free DNA ends caused by DNA damage were detected by labeling with fluorescein-dUTP by thymidine deoxynucleotidyl transferase (TdT) using the detection kit from Roche-Merck (Darmstadt, Germany) (ref. 11684795910) according to the manufacturer protocol. Cells were fixed, permeabilized and blocked according to the section on immunofluorescence.

### 4.11. In vitro Protein Interaction (Pull-Down Assays)

Pull-down assays were performed to study the interaction between VRK1 and SIRT2, in manner similar to previous studies [57]. For this purpose, purified GSTVRK1 and His-SIRT2 in the amounts indicated in the experiment were used.

Proteins were incubated in a buffer containing 20 mM Tris-HCl pH 7.5, 5 mM MgCl_2_, 0.5 mM DTT and 150 mM KCl in a volume of 25 µL at 37 °C and gentle agitation for 45 min. After that, 40 µL of Glutathione Sepharose 4B beads (GE Healthcare; Chicago, IL, USA), previously equilibrated with the same buffer, were added. The mix was incubated overnight at 4 °C. The pull-down was performed by centrifugation (500× *g*, 2 min, 4 °C) and the resin was washed in the same pull-down buffer three times. Agarose-immune complexes were resuspended in sample loading buffer and detected by Coomassie Blue staining (3 g/L Coomassie Brilliant Blue R250, 45% methanol, and 10% glacial acetic acid) in the case of purified proteins.

### 4.12. In vitro Kinase Assay

Reactions were performed in kinase assay buffer (20 mM Tris-HCl pH 7.5, 5 mM MgCl_2_, 0.5 mM DTT, and 150 mM KCl) containing 1 µg of GST-VRK1 wildtype or GST-VRK1-K179E (kinase-dead) and the varying amounts of His-SIRT2, GST-p53(1-84) and recombinant human histone H3 [58]. ATP (10 µM) was added to the mix, in the presence of 7.5 µCi of γ-32P when there was not an available commercial phospho-specific antibody. Reactions were performed at 37 °C for 45 min and stopped by the addition of sample loading buffer. Electrophoresis in acrylamide gel was performed following the aforementioned instructions. When a commercial phospho-specific antibody was available, the signal was detected following immunoblots description. In the case of radioactively labeled membranes, radioactive signal was detected using Fuji Medical X-ray films. Afterwards, membranes were blocked in milk for 1 h at room temperature and incubated with the corresponding primary antibody for 2–4 h. Subsequently, membranes were washed 3 times in TBS-T and incubated with secondary antibodies (ECL Anti-Mouse or Rabbit) for 1 h. Right after 3 more washes, blots were developed with the ECL detection system (Solution A: 0.1 M Tris HCl pH 8.5, 0.2 mM coumaric acid, and 1.25 mM Luminol; Solution B: 3% H_2_O_2_) after 5 min incubation, using Fuji Medical X-ray films.

### 4.13. Statistical Analysis

Graphs and statistical differences were computed using GraphPad Prism 8. Results are presented as dot plots with the median, first and third quartiles and whiskers. After confirming samples did not adjust to a normal distribution (nonparametric distributions) according to a two-tailed Kolmogorov test, a Kruskal–Wallis test was used for two-group comparisons in all experiments. Values of *p* < 0.05 were considered significant. Values of *p* < 0.05 were ranked as *: *p* < 0.05, **: *p* < 0.01 and ***: *p* < 0.001. n.s.: non-significant differences.

## 5. Conclusions

In this work, we have studied the crosstalk between the VRK1 chromatin kinase and the SIRT2 histone deacetylase that regulate the level of H4K16ac, a key modification regulating the accessibility of chromatin. VRK1 promotes the acetylation of H4K16 by activating Tip60, and SIRT2 removes this modification. In order to carry out this role SIRT2 forms as complex with VRK1, inhibiting its activity on Tip60/KAT5, and thus facilitating H4K16 deacetylation. The novel VRK-IN-1 inhibitor facilitates H4 deacetylation and promotes the accumulation of DNA breaks, but prevents the progression of the DNA repair processes. This VRK1 inhibitor can be of use for designing novel synthetic lethality strategies to promote tumor cell death.

## Figures and Tables

**Table 1:** Inhibitors/drug treatments and conditions.

Reagent	Target	Concentration and Time	Reference	Supplier
AGK2	SIRT2	5 µM/24 h	S7577	SelleckChem
AK7	SIRT2	8 µM/24 h	S5914	SelleckChem
Thiomyristoyl	SIRT2	5 µM/24 h	S8245	SelleckChem
MG149	KAT5 (TIP60)	1 µM/24 h	1785	Axon MedChem
VRK-IN-1	VRK1	0–600 nM/24 h	HY-126542	MedChemExpress
Doxorubicin	Intercalates DNA Top2A inhibitor	3 µM/2 h	25316-40-9	Sigma-Aldrich

**Table 2:** Primary antibodies.

Antibody	Type	Dilution (WB/IF)	Clone, Reference	Supplier
53BP1	Rabbit polyclonal	1:1000/1:400	NB100-304	Novus Biologicals
β-actin	Murine mAb	1:2000/-	AC15, A5441	Sigma-Aldrich
Flag Tag	Murine mAb	1:1000/-	M5, F4042	Sigma-Aldrich
Flag Tag	Rabbit polyclonal	1:1000/-	F7425/ab1162	Sigma-Aldrich/ Abcam
GST Tag	Murine mAb	1:1000/-	B-14, Sc-138	Santa Cruz Biotech.
HA.11 Tag	Murine mAb	1:1000/1:1000	901514,16B12	BioLegend
HA Tag	Rabbit polyclonal	1:1000/1:1000	H6908	Sigma-Aldrich
His Tag	Murine mAb	1:1000/-	HIS-1, H1029	Sigma-Aldrich
H3-T3ph	Rabbit polyclonal	1:1000/-	07-424	Millipore
H3	Rabbit polyclonal	1:1000/-	9715	Cell signaling
γH2AX	Murine mAb	-/1:500	JBW301, 05-636	Millipore
H2AX	Rabbit polyclonal	1:1000/-	2595S	Cell Signaling
H4K16ac	Rabbit monoclonal	1:500/1:1.000	Ab109463	Abcam
P53-T18ph	Rabbit polyclonal	1:1000/-	2529	Cell signaling
P53	Murine mAb	1:1000/-	DO-1, Sc-126	Santa Cruz Biotech
VRK1	Murine mAb	-/1:1000	1B5	[54]
VRK1	Murine mAb	1:1000/-	1F6	[54]
VRK1	Rabbit polyclonal	1:1000/-	VC	[54]

**Table 3:** Secondary antibodies.

Antibody	Application/ Dilution	Reference	Supplier
Cy^TM^5-Goat Anti-Mouse	IF 1:1000	115-175-146	Jackson ImmunoResearch
Cy^TM^3-Goat Anti-Mouse	IF 1:1000	15-165-146	Jackson ImmunoResearch
Cy^TM^2-Goat Anti-Rabbit	IF 1:1000	111-225-144	Jackson ImmunoResearch
Sheep ECL Anti-Mouse IgG, Peroxidase Conjugated	WB 1:10,000	NA931	Cytiva-Amersham
Goat ECL Anti-Rabbit IgG, Peroxidase Conjugated	WB 1:10,000	A0545	Sigma-Aldrich
Goat anti-Mouse IgG DyLight 680	WB 1:10,000	35518	Thermo-Fisher
Goat anti-Rabbit IgG DyLight 800	WB 1:10,000	35571	Thermo-Fisher

## Data Availability

Not applicable.

## Supplementary Materials

The following supporting information can be downloaded at: https://www.mdpi.com/article/10.3390/ijms24054912/s1.
