# Supplementary material for: VRK1 Kinase Activity Modulating Histone H4K16 Acetylation Inhibited by SIRT2 and VRK-IN-1
Source: Int J Mol Sci. 2023 Mar 3;24(5):4912. doi: 10.3390/ijms24054912 (PMC10003087; doi:10.3390/ijms24054912)
Supplement: Supplementary file 1 [file ijms-24-04912-s001.zip › Supplementary Figure S1.pdf]

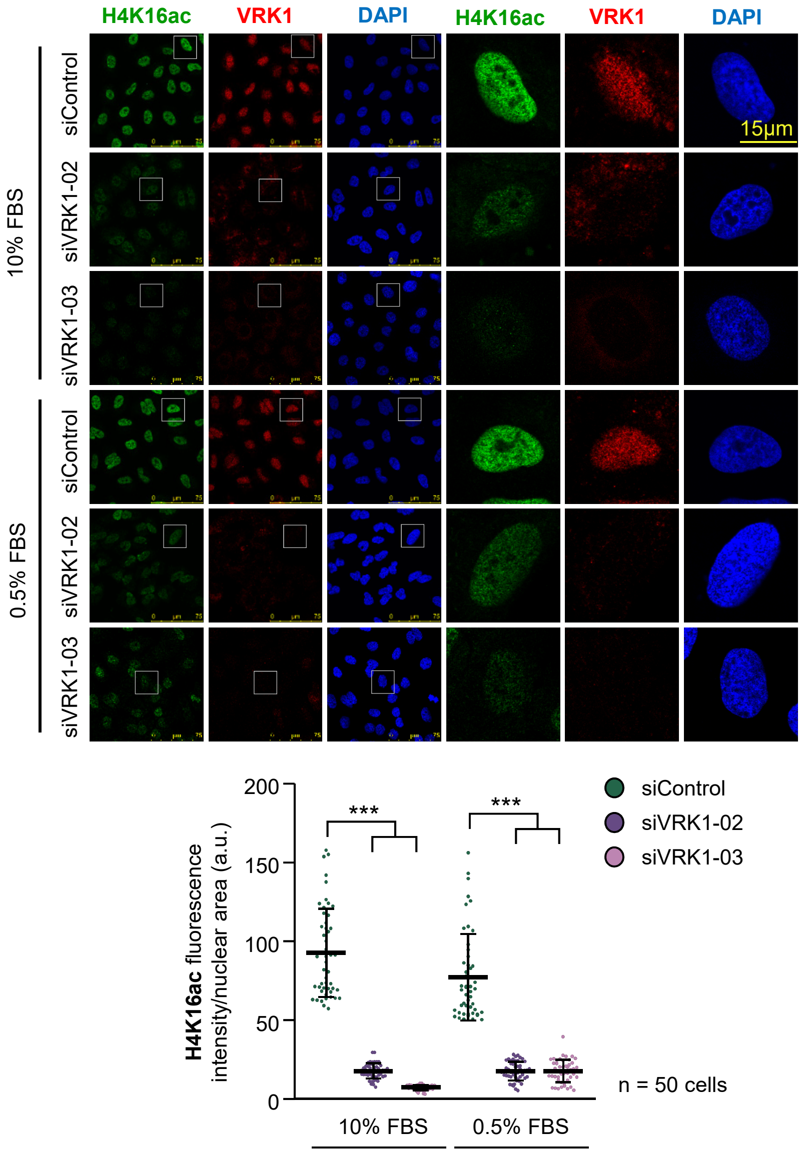

Figure S1. Effect of VRK1 depletion on the levels of H4K16ac induced by doxorubicin treatment. The experiment was performed either in the presence of 10 % serum, or in serum deprived cells (0.5%). The effect of VRK1 depeltion was determined using two different siRNA independently. *** P < 0.001
