# Supplementary material for: VRK1 Kinase Activity Modulating Histone H4K16 Acetylation Inhibited by SIRT2 and VRK-IN-1
Source: Int J Mol Sci. 2023 Mar 3;24(5):4912. doi: 10.3390/ijms24054912 (PMC10003087; doi:10.3390/ijms24054912)
Supplement: Supplementary file 1 [file ijms-24-04912-s001.zip › Supplementary Figure S2.pdf]

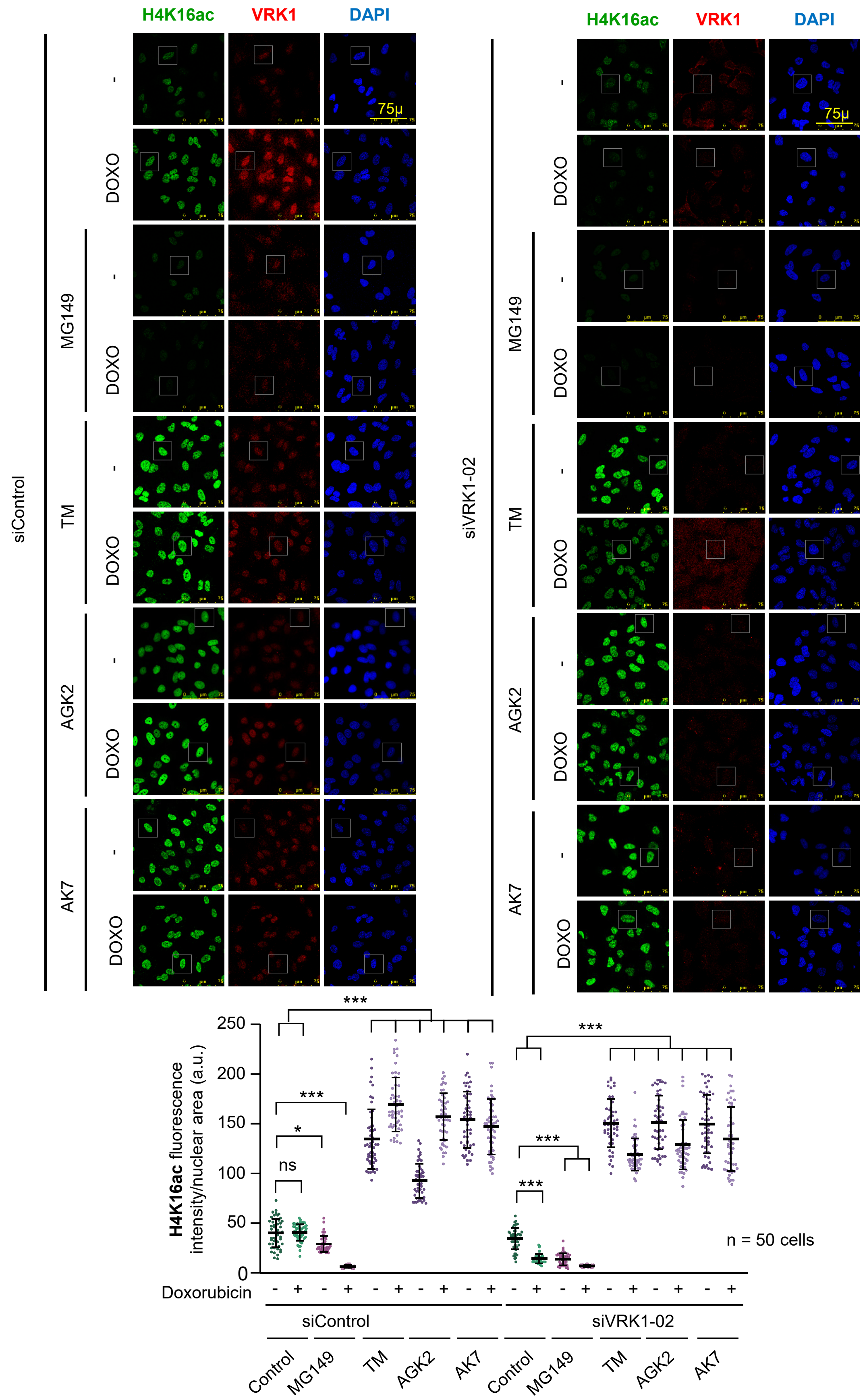

Figure S2. Effect of Tip60 and SIRT2 inhibitors on the H4K16ac levels in the response to doxorubicin. Left: Effect of inhibitors in control cells with endogenous VRK1. Right: Effect of inhibitors in cells depleted of VRK1. ns: not significant. * $P < 0.05$; *** $P < 0.001$
