# Supplementary material for: VRK1 Kinase Activity Modulating Histone H4K16 Acetylation Inhibited by SIRT2 and VRK-IN-1
Source: Int J Mol Sci. 2023 Mar 3;24(5):4912. doi: 10.3390/ijms24054912 (PMC10003087; doi:10.3390/ijms24054912)
Supplement: Supplementary file 1 [file ijms-24-04912-s001.zip › Supplementary Figure S3.pdf]

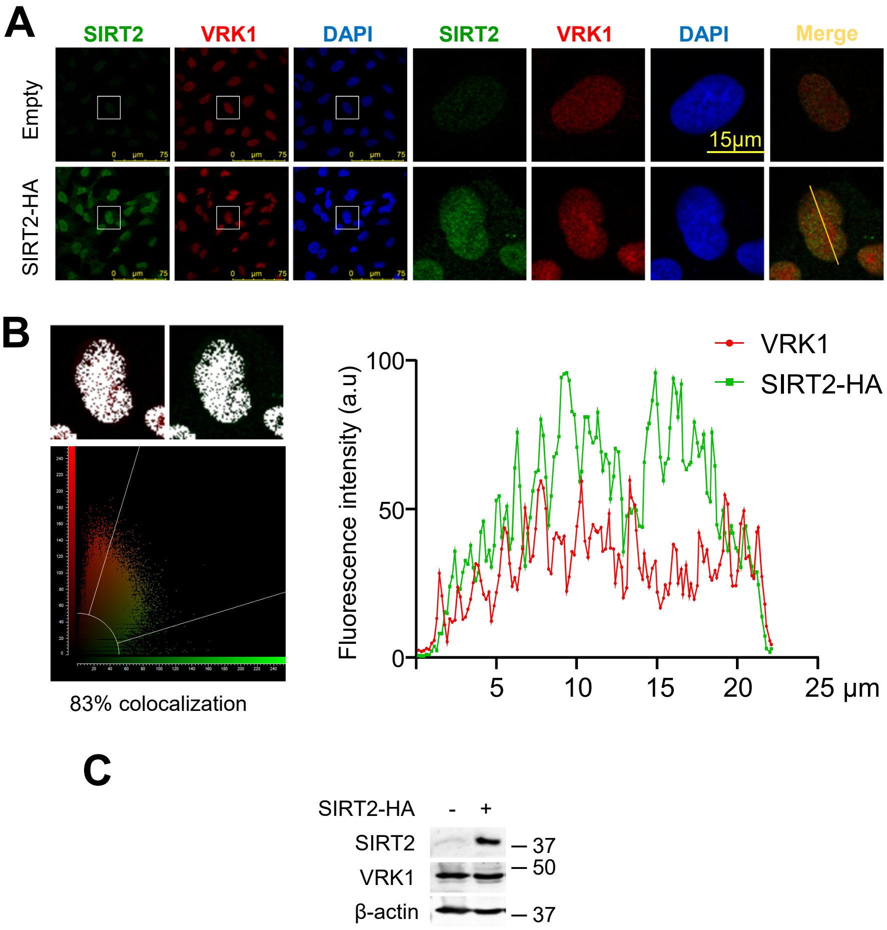

Figure S3. Nuclear colocalization of VRK1 and SIRT2 proteins. A. Colocalization of endogenous VRK1 and transfected SIRT2-HA in A549 cells. Field images are shown to the left. The selected individual cell to show detail is marked by a box and shown to the right. **B.** Overlap of the the immunofluorescence signals of VRK1 (red) and SIRT2 (green) along the plane (line) indicated in the merge image at the right. **C.** Immunoblot showing the expression of VRK1 and SIRT2.
