# Supplementary material for: VRK1 Kinase Activity Modulating Histone H4K16 Acetylation Inhibited by SIRT2 and VRK-IN-1
Source: Int J Mol Sci. 2023 Mar 3;24(5):4912. doi: 10.3390/ijms24054912 (PMC10003087; doi:10.3390/ijms24054912)
Supplement: Supplementary file 1 [file ijms-24-04912-s001.zip › Supplementary Figure S4.pdf]

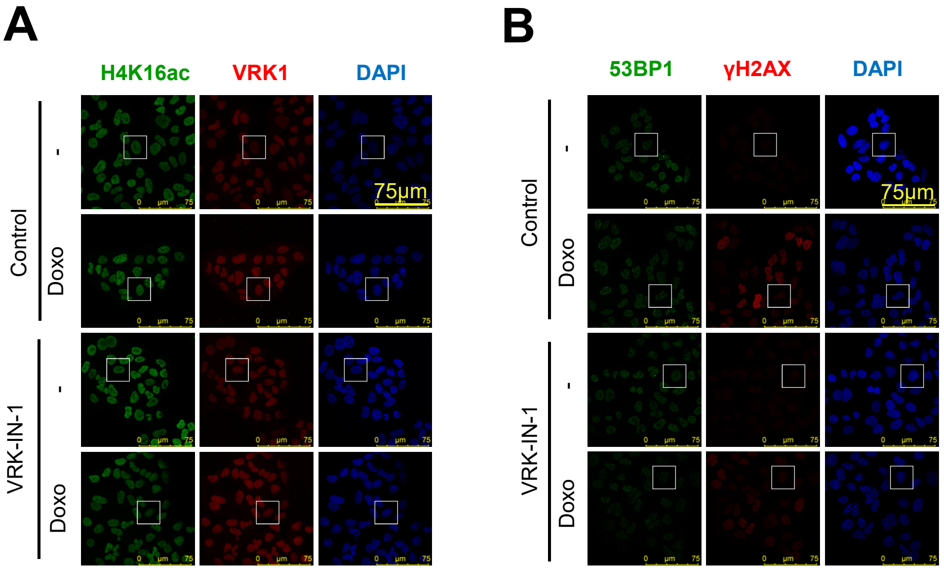

Figure S4. Effect of the VRK-IN-1 inhibitor on the level of H4K16ac (**A**) and 53BP1 foci (**B**) induced in the response to doxorubicin tratment. The boxed cells and the quantification are shown in Figure 7.
